# The impact of butyrate on glycemic control in animals and humans: a comprehensive semi-systemic review

**DOI:** 10.3389/fnut.2025.1603490

**Published:** 2025-06-10

**Authors:** Nouhaila Hamari, Ellen E. Blaak, Emanuel E. Canfora

**Affiliations:** Department of Human Biology, Institute of Nutrition and Translational Research in Metabolism (NUTRIM), Maastricht University Medical Center +, Maastricht, Netherlands

**Keywords:** butyrate production and administration, microbial phenotype, glucose homeostasis, insulin resistance, obesity

## Abstract

The gut microbiome has been identified as a significant factor in host metabolism, playing a key role in the etiology of obesity, type 2 diabetes and cardiometabolic risk. Butyrate, produced by the gut microbiome from indigestible carbohydrates, has been shown to have beneficial effects on body weight control, inflammation, and insulin resistance, primarily evidenced by animal studies and *in vitro* experiments. However, translating these benefits to humans remains challenging due to variability in mode of butyrate administration or production upon fermentation of dietary fibers, as well as in butyrate absorption, and its metabolism. For instance, oral butyrate supplementation can directly increase circulating butyrate levels, thereby targeting peripheral tissues. In contrast, butyrate produced by the gut microbiome may also influence metabolism through local signaling mechanisms affecting peripheral tissues. Additionally, there may be large heterogeneity in the response of the individuals to butyrate interventions. Future research should aim to better understand butyrate kinetics and dynamics and its mechanisms in regulating intestinal and metabolic health. In human studies, longer-term, placebo-controlled trials are needed to establish the efficacy of either targeting butyrate production or supplementation in individuals with obesity and/or metabolic disturbances. Personalized dietary interventions based on individual microbiota composition and/or function and metabolic profiles may optimize butyrate production and its metabolic benefits. This could pave the way for effective butyrate-based interventions to improve metabolic health and prevent obesity-related complications.

## Introduction

The increasing prevalence of metabolic disorders worldwide is primarily attributed to the globalization of the Western lifestyle, marked by shifts toward energy-dense, low-fiber diets, and sedentary habits (1, 2). Obesity and its co-morbidities have become a serious global health and socioeconomic problems of the twenty-first century (3). Currently, one-third of the world’s population is overweight or obese. Moreover, it is expected that half of the world’s population will be overweight or obese by 2030 (4).

Obesity develops in a state of persistent positive energy balance when caloric intake exceeds energy expenditure, leading to weight gain (3). This energy imbalance may be associated with factors such as disrupted appetitive hormone signaling and increased adiposity (5). Over time, the adipose tissue becomes inflamed and losses its capability for storing excess energy as triacylglycerols. This dysfunction leads to ectopic fat deposition in organs such as skeletal muscle and liver (3). Elevated systemic free fatty acids and triacylglycerol concentrations can result in intracellular lipid accumulation, including increased bioactive lipid metabolites that may negatively interfere with insulin signaling (6). Consequently, obesity increases at least six-fold the risk for developing type 2 diabetes (T2DM), depending on individual and population-specific factors (7). Obesity is the main driver of insulin resistance that is observed in T2DM in metabolically active tissues such as adipose tissue, the liver and skeletal muscle. Prediction models estimate that more than 300 million individuals have T2DM as a consequence of obesity by 2025 (8). Moreover, diabetes and obesity are associated with increased vascular stiffness and atherosclerosis, which elevate the risk of developing cardiovascular disease (9). Lastly, excessive body weight is also associated with mental health disorders, with two out of five people who are overweight or obese being diagnosed (10). Hence, there is an urgent need to address the obesity and its related complications pandemic.

Over the past two decades, the gut microbiome has emerged as a critical regulator of host energy and substrate metabolism (3, 4, 11–13). Therefore, the gut microbiota may significantly contribute to the etiology of obesity, insulin resistance and T2DM. One of the important roles of the gut microbes, mainly of anaerobic bacteria, is the fermentation of indigestible carbohydrates, such as dietary fibers, into short-chain fatty acids (SCFA). The most abundant SCFA in the gut are acetate, butyrate and propionate. SCFA are believed to mediate both the gut and host homeostasis by activating the G-protein coupled cell surface receptors (GPRs) such as GPR41, GPR43, and GPR109A, expressed in various tissues including the gut, adipose tissue, skeletal muscle and liver (3, 10). In the gut, SCFA can induce the production of the satiety hormones glucagon-like peptide 1 (GLP-1) and peptide YY (PYY) by interacting with enteroendocrine L-cells. Via signaling or other mechanisms, SCFA may reduce chronic low-grade systemic and tissue inflammation, increase muscle and liver fat oxidation, and affect the efficiency of the adipose tissue to store lipids (3, 10). As a result, SCFA may control a variety of metabolic processes including energy balance, glucose homeostasis, insulin sensitivity and lipid metabolism (9–11).

In particular, the SCFA butyrate seems to serve as the connection between gut microbes and host gastrointestinal and metabolic health (5, 14, 15). In a large cohort of normoglycemic individuals, using genome-wide association summary statistics, higher gut-derived butyrate was linked to enhanced insulin response and improved beta-cell function during a meal test (16). Metagenomic data consistently revealed in multiple independent human cohorts a reduction of butyrate-producing bacteria in individuals with T2DM (16–20). In high fat fed animals and cell-based models, sodium butyrate has been reported to have beneficial effects on body weight control, inflammation and insulin resistance (21–27) by affecting energy expenditure, fat oxidation, and gut integrity. Lastly, some studies on high fat-fed mice have shown that sodium butyrate supplementation reduced food intake by affecting regions in the hypothalamus that regulate food intake, potentially promoting satiety through modulation of the gut-brain neural circuitry (28–30).

Overall, there is supporting evidence that butyrate plays a significant role in energy and glucose homeostasis, and that its production may be diminished in individuals with obesity or metabolic disturbances. Therefore, restoring butyrate-producing bacteria and/or increasing intestinal or systemic butyrate levels through oral supplementation or modulation of the gut microbiota in individuals with overweight and disturbed glucose homeostasis may have beneficial effects on cardiometabolic health and could be of clinical significance. However, it remains uncertain whether the beneficial effects of butyrate supplementation observed in animal models can be effectively translated to humans, given the variations in metabolic, microbial, and lifestyle-related factors among individuals, as well as the differences between the murine and human microbiomes. Additionally, the various approaches to increase butyrate levels in the circulation and/or to stimulate butyrate-producing microbial species might influence metabolic responses differently. Administrating butyrate though oral supplementation or indirectly via stimulation of microbial butyrate producing pathways, such as through prebiotics, may result in distinct pathways for regulating metabolic health (31).

In this review, we use a semi-systematic approach to identify and summarize available literature on studies that have measured either butyrate levels in plasma and/or feces and/or have quantified butyrate producers in relation to markers of glucose homeostasis and/or inflammation in both animals and humans with a particular focus on randomized clinical trials. We further discuss these studies to clarify the mechanisms underlying the action of butyrate action and its relationship to metabolic and intestinal health in both healthy individuals and those with an impaired glucose metabolism. Finally, we address the current butyrate supplementation methods and what is required to improve the therapeutic potential of butyrate supplementation for prevention and treatment of obesity and T2DM.

## Methods

A semi-systematic search was conducted within PubMed. The search strategy was developed through internal discussion within the research team. Literature was managed using EndNote 21. The search strategy consisted of one or a combination of the following search terms using the “AND” and “OR” operators.

For the human studies: “Butyrate [TIAB], “glucose” [TIAB], “insulin” [TIAB], “microbiome” [TIAB], “species” [TIAB] with as filter clinical trial and randomized clinical trial.

For the animal studies: “Butyrate” [TIAB], “glucose” [TIAB], “insulin” [TIAB], “animal” [TIAB], “mice” [TIAB], “rat” [TIAB].

From the identified articles, the titles and abstracts were assessed, and if considered relevant for the present review, the full text of the article was examined in detail (Supplementary Table 1). The focus was on human studies. Only studies that measured either plasma or fecal butyrate levels, and/or assessed butyrate-producing species as well as reported markers of glucose homeostasis (e.g., fasting glucose, postprandial glucose) and/or insulin sensitivity (e.g., HOMA-IR) were included. Inflammatory indicators such as plasma cytokines were also considered if they were reported. Accordingly, prebiotics (e.g., fibers), probiotics (e.g., *Anaerobutyricum soehngenii*), postbiotics (e.g., SCFA, vitamins) and/or high-fiber dietary patterns (e.g., Mediterranean diet) were selected as interventions intending to modify butyrate levels. In addition, additional records were identified though searching reference lists of published manuscripts. Only articles written in English were included.

## Mechanistic insights of butyrate from animal studies

Originally used in livestock farming, butyrate supplementation is now also applied in animal models to study its impact on human diseases. We focused on animal models representing metabolic disorders and with the search string identified in total of 18 animal studies that measured markers of glucose homeostasis and/or inflammation (Table 1). Most of the studies used 5% weight/weight sodium butyrate (NaBut) as supplementation form in male C57BL/6J mice, with the number of mice per group ranging from 5 to 14 (22, 23, 25, 26, 29, 32–34). Additionally, male Sprague–Dawley and female Wistar rats were used in some studies, with group sizes ranging from five to seven per group (35–39). Overall, butyrate counteracted the hyperglycemia and hyperinsulinemia induced by a high-fat diet (HFD), resulting in reductions in both fasting and postprandial glucose and insulin responses (22, 23, 26, 28, 29, 32–36, 38, 40, 41). Occasionally, these concentrations returned to normal levels resembling those seen in control groups fed a chow diet (25, 35, 41–43). Interestingly, in three studies, a control group consisting of lean mice on a chow diet also received NaBut, but no improvements in glucose homeostasis were observed (29, 40, 43). These rodents’ studies have provided mechanistic insights though which butyrate exerts its beneficial effects on glucose homeostasis when animals are exposed to an HFD, which will be further discussed below.

**Table 1:** The animal studies on oral butyrate supplementation assessing glucose homeostasis and/or insulin sensitivity markers.

Animal studies	Breed	Type of intervention	Glucose homeostasis	Insulin sensitivity	Inflammation
Gao et al. 2009 (22)	C57BL/6J mice	12 wks 5%wt/wt of NaBut	↓ fasting glucose	↑ IS	N.M.
Badejogbin et al. 2019 (35)	Wister rats	6 wks NaBut (200 mg/kg)	↓ post load glycaemia	↓ HOMA-IR, Fasting insulin	N.M.
Jiao et al. 2021 (32)	C57BL/6J mice	35 d 5% NaBut	↓ serum glucose	N.C.	↓ IL-1β
Raso et al. 2013 (36)	Sprague–Dawley rats	4 wks NaBut (20 mg/kg) with HFD	↓ glucose tolerance	↓HOMA-index, Fasting insulin	↓ hepatic inflammation (TNF-α)
Li et al. 2018 (28)	APOE*3 mice	9 wks 5% w/w NaBut	Tendency lower plasma glucose	↓ HOMA-IR, Fasting insulin	N.M.
Gao et al. 2019 (26)	C57BL/6J mice	16 wks NaBut (400 mg/kg)	↓ glucose tolerance	↓ IR	N.M.
Mollica et al. 2017 (23)	C57BL/6J mice	12 wks NaBut (100 mg/kg)	↓ glucose tolerance	↓ HOMA-IR, Fasting insulin	↓ proinflammatory serum markers
Zhang et al. 2017 (40)	CD1-mice	12 wks NaBut (1% in water)	↓ fasting glucose	↓ serum insulin	N.M.
Sun et al. 2019 (39)	Sprague–Dawley rats	7 wks NaBut (300 mg/kg)	↓ postprandial glucose	↑ insulin signaling pathway	↓ oxidative stress
Tang et al. 2022 (37)	Sprague–Dawley rats	12 wks NaBut (4%/5%/6%)	↓ blood glucose	↓ IR index	N.M.
Henagan et al. 2015 (25)	C57BL/6J mice	10 wks NaBut (5% w/w)	↓ glucose tolerance	↓ insulin tolerance	N.M.
Khan et al. 2016 (38)	Sprague–Dawley rats	10 wks NaBut (400 mg/kg)	↓ plasma glucose, HbA1c	↓ IR	N.M.
Pedersen et al. 2023 (42)	db/db mice	5 wks butylated starch	↓ postprandial glucose	↓ HOMA-IR, plasma insulin	↓ inflammation markers of AT and liver
Aguilar et al. 2018 (43)	ApoE KO mice	10 wks NaBut (10 mL/kg)	↓ glucose tolerance	↑ IS	N.M.
Zhou et al. 2018 (33)	C57BL/6J mice	16 wks (200 mg/kg)	↓ serum glucose	↓ fasting serum insulin	↓ liver inflammation
Zhu et al. 2022 (34)	C57BL/6J mice	14 wks NaBut (0.4% w/w)	↓ fasting glucose	↓ HOMA-IR	↓ TNF-α
Matheus et al. 2017 (29)	C57BL/6J mice	8 wks NaBut (5% w/w)	↓ glycemia	↓ IR	N.C.
Fu et al. 2023 (41)	C57BL/6J mice	8 wks NaBut (200 mg/kg)	↓ glucose tolerance, fasting glucose	↓ insulin tolerance, fasting insulin	N.M.

IS, Insulin sensitivity; IR, Insulin resistance; HOMA-IR, Homeostatic model assessment for insulin resistance; N.C., no change; N.M, not measured.

### Butyrate and body weight control

Obesity and central adiposity are strongly linked to chronic diseases, while modest weight loss (between 5 and 10%) has been shown to improve glucose homeostasis in individuals with obesity (44, 45). Numerous animal studies (22, 23, 25, 26, 28, 29, 37, 39–41, 43) have reported a decrease in body fat content and lower body weight after NaBut supplementation (commonly at 5% weight/weight for 10–12 weeks) following a HFD. An explanation for the improved body composition could be due to enhanced fatty acid oxidation, supported by higher levels of energy expenditure and oxygen consumption, along with lower respiratory exchange ratio in NaBut treated mice (22, 23, 28, 29, 41). Butyrate supplementation also improved glucose tolerance and insulin signaling in these animal studies (22, 23, 25, 28, 29, 32, 34–37, 40, 41, 46).

### Butyrate and skeletal muscle function

Skeletal muscle is the primary site of glucose uptake and a characteristic of the insulin resistant muscle may be a reduced oxidative capacity and mitochondrial dysfunction (47). It appears from a number of studies that NaBut has the potential to mitigate insulin resistance by enhancing skeletal muscle function through its effects on mitochondrial function.

Three studies in C57BL/6J mice (22, 25, 32) reported that butyrate induced type I fiber differentiation and higher abundance of oxidative fibers in skeletal muscle, as supported by increased protein expression of type I myosin heavy chain (MyHC) and myoglobin (22). They also observed an upregulation of key mitochondrial transcription factors such as peroxisome proliferator-activated receptor-alpha gamma coactivator (PGC-1α) and peroxisome proliferator-activated receptor gamma (PPAR-δ), in both skeletal muscle tissue and L6 cells treated with NaBut. These molecular changes were accompanied by reduced glucose levels and improved insulin sensitivity. The studies administrated 5% of NaBut supplementation to male C57BL/6J mice upon HFD-feeding for either 5 weeks (32), 10 weeks (25), or 12 weeks (22). PGC-1α is a crucial transcriptional coactivator that regulates mitochondrial and metabolic processes involved in cellular metabolism, including both carbohydrate and lipid metabolism (48). This coactivator also stimulates multiple other transcription factors, including PPAR-δ in the muscle, leading to an increase in the expression of insulin-dependent glucose transporter 4 (GLUT-4). In turn, this can result in greater glucose uptake into the muscle, thereby reducing plasma glucose levels (49). Furthermore, in mice treated with NaBut, the muscle exhibited higher levels of AMP-activated protein kinase (AMPK) and p38, both of which are kinases known to phosphorylate PGC-1α (50). AMPK, in particular, serves as a critical regulator of cellular metabolism mediating various pathways in the muscle such as glucose uptake, mitochondrial biogenesis, and fatty acid oxidation (51). Butyrate may activate AMPK through multiple pathways. First, upon cellular uptake via monocarboxylate transporters 1 and 4 (52), which are expressed in muscle, liver, intestine and adipose tissue, butyrate is metabolized in mitochondria, increasing the AMP: ATP ratio and activating AMPK. Second, butyrate binds to GPRs, particularly GPR41, which may activate AMPK indirectly by lowering intracellular cAMP levels, thus relieving the inhibitory effect of protein kinase A on AMPK (53). Third, butyrate’s inhibition of histone deacetylases (HDACs), which are enzymes that repress gene expression by removing acetyl groups from histones promoting chromatin condensation, can enhance the transcription of PPAR-δ target genes (54). This promotes oxidative metabolism, further increases the AMP: ATP ratio, and activates AMPK and PGC-1α signaling pathways. The inhibition of HDACs by butyrate specifically in skeletal muscle has been confirmed both *in vitro* and *in vivo* (22, 54). Through AMPK activation, butyrate may also inhibit the mammalian target of rapamycin (mTOR) pathway (55), a key regulator of cell growth and metabolism in response to amino acids and insulin. mTOR inhibition, potentially reinforced by HDAC inhibition affecting its upstream regulators such as IRS-1, PI3K and Akt, can lead to enhanced autophagy, improved mitochondrial quality control, reduced lipid accumulation, and improved insulin sensitivity.

Taken together, these findings suggest that butyrate enhances skeletal muscle metabolism and insulin sensitivity through coordinated effects on mitochondrial function, AMPK activation and downstream effects and modulating of fiber type composition.

### Butyrate and adipose tissue function

An important organ regulating whole-body energy, glucose homeostasis, and lipid metabolism is the adipose tissue (56). The mitochondria of adipocytes regulate key processes including adipocyte differentiation (e.g., PPAR-γ), oxidative capacity, insulin sensitivity (e.g., GLUT-4), and browning/adaptive thermogenesis (e.g., PGC-1α) (57). Studies in C57BL/6J mice and Sprague Dawley rat (32, 34, 36) reported increased expression of mitochondrial biogenesis proteins nuclear respiratory factor 1 (NRF-1), and mitochondrial transcription factor A (Tfam), alongside PGC-1α and PPAR-γ, in white adipose tissue of rodents treated with NaBut ranging from 2 to 5 weeks. Another mechanism contributing to improved adipose tissue insulin sensitivity involves the upregulation of GLUT-4 expression (26, 43) and enhanced secretion of adiponectin (43). Interestingly, NaBut affects adipocyte size and expansion, leading to reduced hypertrophy and an increased number of adipocytes per unit area (38, 43). These findings collectively suggest that butyrate beneficially modulates adipose tissue morphology and function, leading to improved insulin sensitivity.

### Butyrate in gut intestinal health and inflammation

Given the association between low-grade inflammation and the development of metabolic diseases (58), mitigating inflammation could potentially enhance glucose homeostasis. Mattace Raso et al. (36) investigated the anti-inflammatory effects of NaBut over 4 weeks in a rat model of hepatic steatosis and insulin resistance induced by an HFD. They discovered that butyrate supplementation reduced hepatic expression of tumor necrosis factor-α (TNF-α), monocyte chemoattractant protein 1 (MCP-1), Interleukin (IL)-1β, IL-6, and inhibited the activation of nuclear factor kappa B (NF-κB) and hepatic Toll-like receptors pattern signaling in the liver. In line with these results, Mollica et al. (23) demonstrated that 6 weeks of NaBut supplementation decreased inflammation, as evidenced by reduced concentrations of several HFD-induced increases in serum pro-inflammatory markers in the systemic circulation such as TNF-α, MCP-1 and IL-1β. These findings align with the assumption that butyrate lowers serum cytokines (32, 34, 42). Additionally, Mollica et al. (23) and Zhu et al. (34) observed a decline in plasma lipopolysaccharide (LPS), indicating the anti-inflammatory properties of butyrate. The suppressing of NF-κB by butyrate has been demonstrated in multiple metabolic tissues such as liver, adipose tissue, intestinal epithelium, either through inhibition of IκB kinase (IKK), thereby preventing NF-κB nuclear translocation, or via HDAC inhibition, which reduces transcription of inflammatory genes (59). As NF-κB is a key regulator of inflammation, its inhibition by butyrate may lead to reduced systemic inflammation and enhanced intestinal barrier integrity (reduced metabolic endotoxemia). In line, Wanjun et al. (27) found that HFD feeding significantly reduced tight junction proteins in the intestinal epithelium, whereas NaBut restored the expression of tight junction proteins in both ileum and colon, accompanied by reduced inflammation and plasma LPS levels. Both Matheus et al. (29, 60) and Zhu et al. (34) found that NaBut significantly reduces intestinal permeability upon HFD-feeding by restoring alterations in the content of the tight junction proteins in intestinal permeability cells. Overall, butyrate was able to prevent both systemic and liver inflammation by regulating the production of inflammatory molecules and enhancing intestinal barrier function.

Finally, animal studies have also demonstrated the relevance of butyrate-producing species in supporting intestinal and immune health and metabolic health. Li et al. (61) showed that fecal microbiota transplantation from lean mice fed a HFD with butyrate to antibiotic-treated mice reduced HFD-induced weight gain and improved insulin resistance. The beneficial metabolic outcomes were accompanied by an increase in relative abundance of *Lachnospiraceae* bacterium, which was negatively correlated with multiple metabolic parameters. In addition, a decrease of Bacteroides was observed (61), indicating a potential role of NaBut-induced microbial changes in metabolic benefits. Another mice study (26) found that a 16-weeks HFD regime caused microbial dysbiosis, with species like p_Lachnospiraceae, p_Rikenellaceae, and p_Paraprevotellaceae and metabolic syndrome-related metabolites such as choline being positively correlated with disturbances in various metabolic parameters. In contrast mice on a HFD with NaBut showed a decreased relative abundances of these taxa along with lower fasting blood glucose, serum insulin levels and body weight (26). The results suggest that butyrate can counteract HFD-induced microbiome dysregulation. Wanjun et al. (27) also reported that NaBut modulated the gut microbiota in HFD-fed mice, reducing the Firmicutes to Bacteroides ratio toward that of the control group, along with reduced systemic inflammation and improved intestinal barrier integrity. Taken together, these findings imply that butyrate indirectly influences body weight and insulin sensitivity by modulating the gut microbiota composition and functionality.

In summary, sodium butyrate counteracts the metabolic disturbances caused by a HFD in animals, primarily observed in male C57BL/6J mice, with an average dose of 5% weight/weight NaBut over a duration of 10 weeks (Table 1). Overall, NaBut positively impacted body weight regulation, glucose homeostasis, and inflammation, influencing metabolic processes across different tissues, although the precise mechanisms of action remain incompletely understood. Potential approaches include targeting AMPK pathways, enhancing intestinal health and barrier integrity, improving mitochondrial function, and inhibiting HDAC. It is important to keep in mind that the doses used in rodent studies could not directly translated to those typically used in butyrate supplement studies or the levels of butyrate that are microbially produced after, e.g., high dietary fiber intake in humans.

## Evidence from humans: clinical trials

For the human studies we identified 35 studies that measured either plasma and/or fecal butyrate levels, and microbial composition and/or activity including butyrate-producing species along with markers of glucose homeostasis (Table 2). These clinical trials can be categorized based on intervention duration (acute, short-term or long-term) and the type of intervention to (indirectly) modulate butyrate levels and microbial activity (prebiotic, probiotic, butyrate supplementation and dietary patterns).

**Table 2:** The human intervention studies measuring microbial butyrate activity and/or butyrate levels with glucose homeostasis and/or insulin sensitivity markers.

Human studies	Study design	Type intervention	Plasma butyrate	Fecal butyrate	Microbiota composition	Glucose homeostasis	Insulin sensitivity	Inflammation
Acute setting								
Nilsson et al. 2010 (65)	RCD Healthy (*n* = 15)	*Cereal-based* meal test vs white wheat flour	↑	N.M.	N.M.	↓ Postprandial glucose ↑ Glucose tolerance	↓ Serum insulin	N.M.
Sandberg et al. 2016 (64)	RCD Healthy (*n* = 19)	*Whole grain rye kernel bread* vs white wheat flour bread	↑	N.M.	N.M.	↓ Blood glucose	↓ Serum insulin	N.C.
Tarini et al. 2010 (62)	RCD Healthy (*n* = 12)	High-fructose corn syrup with and without *inulin*	↑	N.M.	N.M.	N.C.	N.C.	N.M.
Sandberg et al. 2018 (63)	RCD Healthy (*n* = 38)	*Whole grain rye bread* vs white wheat flour	↑	N.M.	N.M.	N.C.	↑ IS index	↓ IL-1β
Canfora et al. 2022 (11)	RCD 1 Lean (*n* = 11) 2 Prediabetic (*n* = 12)	*1 Inulin + RS* *2 Beta-glucan + RS* vs maltodextrin	1 ↑ 2 ↑	1 N.C. 2 N.C.	1 Tendency for microbial shift 2 N.C.	1 ↓ Postprandial glucose 2 N.C.	1 ↑ Postprandial IS 2 N.C.	N.M.
Van der Beek et al. 2018 (74)	RPCC Overweight/obese (*n* = 14)	*Inulin in high-fat milkshake* vs maltodextrin	N.C.	N.C.	N.M.	↓ Plasma early postprandial glucose response	↓ Plasma early + late insulin postprandial response	N.M.
Hartvigsen et al. 2014 (76)	RCD Metabolic syndrome (*n* = 15)	*1 AX* *2 rye kernels* *3 AX with rye kernels* vs wheat porridge	1 ↑ 3 ↑	N.M.	N.M.	3 ↓ Acute glucose response	3 ↓ Acute insulin response	N.M.
Canfora et al. 2017 (68)	RCD Overweight/obese (*n* = 12)	*Rectally infused SCFA mixtures* vs sodium chloride	↑	N.M.	N.M.	N.C.	N.C.	N.C.
Costabile et al. 2023 (73)	RCD Overweight/obese (*n* = 20)	*High amylose rich bread* vs wheat flour	N.C.	N.M.	N.M.	↓ Plasma postprandial glucose profiles	↓ Plasma postprandial insulin response	N.M.
Fernandes et al. 2011 (75)	RCD Normal IS (*n* = 9) IR (*n* = 9)	Glucose drink with and without *inulin*	N.C.	N.M.	N.M.	N.C.	N.C.	N.C.
Short-term								
Liu et al. 2017 (89)	RCD, 2 wks Healthy (*n* = 35)	*FOS* vs *GOS*	N.M.	↓	↓ butyrate-producing bacteria	Worsened OGTT response	N.M.	N.M.
Bouter et al. 2018 (77)	RPCC, 4 wks 1 Lean (*n* = 9) 2 Metabolic syndrome (*n* = 10)	*Oral butyrate*	1 N.C. 2 N.C.	1 N.C. 2 ↓	+ correlation 1 *Bacteroides* & plasma butyrate 2 *Coriobacteriacae* & hepatic IS	N.M.	1 ↑ Peripheral/hepatic IS 2 N.C.	N.M.
Liu et al. 2022 (93)	RPCT, 2 wks Healthy (*n* = 105)	*Riboflavin 50 & 100 mg vs* placebo	N.M.	↑	+ Correlation: Fecal butyrate & Fecal bacterium	N.C.	N.C.	N.M.
Hughes et al. 2021 (86)	RPCC, 1 wks Healthy (*n* = 30)	*Resistant starch type 2* vs control wheat	N.M.	N.C.	+ Correlation: fecal butyrate & butyrate-producing species	↓ Postprandial glucose	↓ Postprandial insulin response	N.M.
Mcintosh et al. 2003 (85)	RPCC, 4 wks Overweight men (*n* = 28)	1 *Whole-grain rye flour* *2 whole grain wheat flour* vs low fiber refined cereal foods	N.M.	1 ↑ 2 N.C.	N.M.	1 ↓ Postprandial glucose response 2 ↓ Postprandial glucose response	1 ↓ Postprandial insulin response 2 Postprandial insulin response	N.M.
Puhlmann et al. 2022 (84)	RPCT, 3 wks Prediabetic (*n* = 55)	*Dried chicory root* vs maltodextrin	N.C.	↑	↑ Anaerostipes spp.	↓ Glucose coefficient of variation	↓ HOMA-IR	N.M.
Giljamse et al. 2020 (91)	PBDT, 4 wks Metabolic syndrome (*n* = 24)	*Live strain of A. soehngenni*	N.M.	N.C.	↑ *Anaerobutyricum* spp.	N.C.	N.C.	N.M.
de Groot et al. 2020 (78)	RPCC, 4 wks Type 1 diabetes (*n* = 30)	*Sodium butyrate* vs placebo capsules	N.M.	↓	Distinctive taxa: Lachnospiracae spp., Ruminococcaceae spp., *Marvinbryantia* spp.	N.C.	N.C.	N.M.
Koopen et al. 2022 (92)	RPCC, 4 wks Overweight/obese & metabolic syndrome (*n* = 12)	*Duodenal Infusion of A. soehngenni cells* vs 10% glycerol	N.M.	N.C.	N.M.	↓ Glucose excursions	N.C.	N.M.
Lee et al. 2017 (90)	RCT, 4 wks Healthy (*n* = 30)	*Bifidobacterium animalis* subsp. *Lactis with and without* yoghurt	N.M.	N.C.	N.M.	N.C.	N.C.	N.C.
Long-term								
Chambers et al. 2019 (102)	RPCC, 6 wks Overweight/obese (*n* = 12)	*Inulin-propionate ester, inulin* vs cellulose	N.C.	N.C.	N.M.	N.M.	↓ HOMA-IR ↑ Matsuda index	N.M.
Mueller et al. 2020 (108)	RPCC, 6 wks Overweight/obese (*n* = 163)	*1 high fiber diet (carbs)* *2 high fiber diet (protein)* *3 high fiber diet (unsaturated fats)*	2 ↑	N.M.	N.M.	N.C.	N.C.	N.M.
Palacios et al. 2020 (106)	RCT, 12 wks Type 2 diabetes (*n* = 60)	*Multi-strain probiotic* vs placebo supplement	↑	N.M.	↑ *Akkermansia muciniphila*, *Bacteroides caccae*	N.C.	N.C.	N.C.
Canfora et al. 2017 (104)	RPCT, 12 wks Overweight/obese & Prediabetic (*n* = 44)	*GOS* vs maltodextrin	N.C.	N.C.	↑ *Bifidobacterium*	N.C.	N.C.	N.C.
Wolever et al. 2000 (96)	RCT, 4 mos Impaired glucose tolerance (*n* = 22)	*Acarbose* vs placebo	↑	N.M.	N.M.	↓ 12 h mean glucose concentration	↓ 12 h mean insulin concentration	N.M.
Perraudeau et al. 2020 (107)	RPCT, 12 wks Type 2 diabetes (*n* = 76)	*Probiotic stains* vs silica	N.M.	N.C.	↑ *Akkermansia muciniphila*, Anaerobutyricium hallii	↓ Total glucose AUC_0-180 min_	N.M.	N.C.
Zhao et al. 2018 (105)	RCT, 12 wks Type 2 diabetes (*n* = 43)	*High fiber diet + prebiotics + acarbose* vs standard care	N.M.	↑	↑ butyrate-producing bacterial strains	↓ Fasting glucose ↓ Postprandial glucose ↓ HbA1c	N.M.	N.M.
Ding et al. 2022 (103)	RCT, 12 wks Type 2 diabetes (*n* = 85)	*Germinated brown rice* vs refined white rice	N.M.	↑	↑ *Bacteroides,* Bifidobacteriales	↓ Fasting glucose ↓ HbA1c	↓ Fasting insulin	↓ IL-6, IL-8, LPS
Upadhyaya et al. 2016 (98)	RPCT, 12 wks Metabolic syndrome (*n* = 20)	*Resistant starch type 4 flour* vs control flour	N.M.	↑	+ Correlation: fecal butyrate & *Ruminococcus lactaris* and Oscillospira spp.	↓ Fasting glucose ↓ HbA1c	N.M.	↓ IL-6, TNF-α
Bell et al. 2022 (97)	OLFT, 12 wks Type 1-diabetes (*n* = 18)	*Type 2 resistant starch*	↑	↑	+ Correlation: fecal butyrate & *Parabacteroides, Bifidobacterium*	N.C.	N.C.	N.M.
Meslier et al. 2020 (110)	RCT, 8 wks Overweight/obesity (*n* = 82)	*Mediterranean diet* vs habitual diet	N.M.	N.C.	↑ Roseburia, Lachnospiraceae, *Faecalibacterium prausnitzii*	N.C.	N.C.	N.C.
Freeland et al. 2009 (100)	RCT, 1 y Hyperinsulinemia (*n* = 28)	*High-wheat fiber cereal* vs low-fiber cereal	↑	N.M.	N.M.	N.C.	N.C.	N.M.
Vitale et al. 2021 (109)	RPCT, 8 wks Overweight/obesity (*n* = 29)	*Mediterranean diet* vs Western diet	N.C. fasting ↑ postprandial	N.M.	N.M.	↓ Plasma glucose response	↓ Plasma insulin response	N.M.
Vetrani et al. 2016 (101)	RCT, 12 wk Metabolic syndrome (*n* = 45)	*Whole-grain cereal vs refined cereal goods* vs refined cereal goods	N.C.	N.M.	N.M.	N.C.	↓ Postprandial insulin response	N.C.
Kjølbæk et al. 2020 (82)	RPCC, 12 wks Metabolic syndrome (*n* = 27)	*AX oligosaccharides*	N.M.	N.M.	↑ butyrate-producing species	N.C.	N.C.	N.M.

N.C., no change; N.M, not measured; AX, Arabinoxylan; RCD, Randomized controlled cross-over; RPCC, Randomized placebo-controlled cross-over; RPCT, Randomized placebo-controlled trial; PBDT, Phase blinded dose trial; RCT, Randomized controlled trial; OLFT, Open-label feasibility trial.

### Acute interventions

In total, we identified 10 clinical trials that investigated the acute effects of dietary fiber-derived butyrate on indicators of glucose metabolism and insulin sensitivity (Table 2). Apart from one study, all interventions used dietary fibers such as whole grain, rye, inulin, amylose and arabinoxylan.

First, the studies conducted in lean and metabolically healthy individuals that examined the effects of dietary fibers will be discussed (Table 2). Tarini et al. (62) showed that inulin (oligo-fiber instant) consumption after 6 h increased postprandial serum butyrate concentration but did not affect glucose and insulin levels. Sandberg et al. (63, 64) reported an increase in plasma butyrate concentration following consumption of whole grain rye kernel bread, which was accompanied with decreased blood glucose (finger-prick) and serum insulin concentrations. Nilsson et al. (65) tested eight different cereal-based evening meals. They found that the meals containing high amylose barley kernels and high β-glucan barley kernels significantly improved glucose tolerance and lowered serum insulin concentrations after a standardized breakfast on the following morning. Furthermore, these breads led to elevated plasma butyrate concentrations both fasting and 30 min after consumption of the standardized breakfast. Interestingly, the increases in butyrate levels were inversely correlated with postprandial glucose response and serum insulin responses. Both types of breads were rich in resistant starch (RS) type 2 and soluble dietary fibers mainly consisting of β-glucans. In line with these findings, previous studies have indicated that β-glucans and RS can stimulate butyrate production upon colonic fermentation (66, 67). Surprisingly, the high amylose bread led to a slightly higher plasma butyrate concentration the next morning after the standardized breakfast compared to the high β-glucan bread (65). This discrepancy may be due to the higher content of viscous dietary fibers in the high-glucan bread, which contains more soluble fibers that could affect the transit time. The dietary fibers content also showed a positive correlation with the morning plasma butyrate concentration (65), suggesting that the bread’s composition (quality of dietary fibers) might affect the delivery of fibers to the distal colon for fermentation which has been associated with a higher release of SCFA and beneficial metabolic effects (11, 68, 69). The latter was demonstrated in the study by Van der Beek et al. (69), where distal colonic infusions of acetate improved fat oxidation and plasma PYY concentration and slightly reduced inflammation markers in normoglycemic men with overweight/obesity, while proximal colonic infusions showed no effects. In line, Canfora et al. (11) demonstrated that in lean individuals, fasting plasma butyrate concentration and breath hydrogen exertion only increased the morning after 1 day supplementation of long-chain inulin + RS type 2, and not with long-chain inulin alone. The increase in circulating butyrate was accompanied by improved postprandial insulin sensitivity. The study also used *in vitro* model of the human colon with pooled fecal microbiota samples form obese, prediabetic and nonglycemic individuals, demonstrating that adding RS to long-chain inulin led to greater cumulative production of acetate, butyrate, and total SCFAs in the distal colon, compared to long-chain inulin alone (11). Slow-fermentable fibers or a mixture of dietary fibers might result in saccharolytic fermentation (breakdown of carbohydrates) throughout the whole colon, including the distal colon. Distal colonic saccharolytic fermentation is linked with improved metabolic health outcomes due to the higher distal colonic density of SCFA receptors, a higher rate of SCFA release into the circulation, and a more diverse microbiome composition (e.g., higher abundance of *Bacteroidetes*) compared to the proximal colon (68–70). Since fermentation is a continuous process, SCFA concentrations can fluctuate over time, with higher plasma SCFA typically reached later in the postprandial phase, as observed in pigs fed various cereal breads (71, 72).

Similarly, studies have investigated the acute effect of dietary fibers in individuals with overweight/obesity and/or are metabolically compromised. Costabile et al. (73) reported significant reductions in postprandial glucose after a breakfast with high amylose (70 and 85%) wheat bread (measurements 4 h after consumption). They also observed a reduced insulin response after a lunch of similar composition (measurements 2 h later) in individuals with overweight and obesity. However, they found a significant increase in plasma propionate, instead of butyrate, 6 h after consuming the breakfast. Although the overall plasma profile (0–360 min) for butyrate did not differ, plasma butyrate was significantly higher at 240 min after consuming the bread containing 70% high amylose compared to the control bread (73). Similarly, Van der Beek et al. (74) found a trend toward higher plasma butyrate after consumption of a high-fat milkshake containing native inulin compared to the placebo milkshake with maltodextrin in individuals with overweight and obesity, along with lower postprandial glucose and insulin responses. Fernandes et al. (75) found no changes in serum butyrate, plasma glucose, and insulin levels for 4 h after ingesting inulin (oligo-fiber instant) in participants that were both insulin sensitive as insulin resistant. Canfora et al. (11) found an increase in plasma butyrate the morning after combined beta-glucan and RS supplementation in individuals with overweight/obesity and prediabetes. However, they did not observe any changes in resting and postprandial substrate metabolism, insulin sensitivity, or satiety hormones. Surprisingly, Hartvigsen et al. (76) observed an increase in postprandial plasma butyrate concentration (at 360 min) after consumption of porridge containing either arabinoxylan or whole grain rye kernels in individuals with metabolic syndrome. This was accompanied by a reduced incremental area under the curve (iAUC) for glucose and insulin within the first 2 h, compared to wheat porridge. Nevertheless, the increased butyrate concentration did not affect glucose, insulin, free fatty acids (FFA), GLP-1 or ghrelin levels after the standardized lunch. Nor were they able to establish a clear relationship between improved glycemic response to arabinoxylan-rich meals and increased SCFA levels. Another study of Canfora et al. (68) rectally infused physiological concentrations of three SCFA mixtures, all with relatively high acetate levels, with each mixture further enriched in either acetate, propionate, and butyrate, in individuals with overweight and obesity. All three mixtures significantly increased fasting circulating butyrate and fasting fat oxidation. The high acetate and propionate mixture also increased fasting plasma acetate concentrations, which were positively correlated with increases in resting energy expenditure and fasting fat oxidation (68). Fasting and postprandial glucose and insulin concentrations did not differ between the treatments.

Based on the acute studies (Table 2), a link between increased plasma butyrate and improved glucose homeostasis is observed in both metabolically healthy individuals and, to a lesser extent, those with obesity and/or metabolic disturbances. Of course, the measurements in these acute studies were conducted over a limited number of hours, making it challenging to translate it into longer term effects. Furthermore, merely studies in healthy individuals were included, making it uncertain to what extent the outcomes translate to metabolically comprised conditions.

### Short-term interventions

Altogether, 11 studies examined the short-term impacts of dietary fibers, postbiotics, and probiotics, potentially influencing butyrate, on markers of glucose homeostasis over durations ranging from 1 to 4 weeks (Table 2).

Bouter et al. (77) investigated the impact of oral sodium butyrate capsules over 4 weeks in both lean individuals and individuals with metabolic syndrome. In both groups, the supplementation did not alter plasma and fecal butyrate concentrations and microbiota composition. However, by applying an algorithm, they identified that the abundances of the bacterial strains *Lachnospiracae* and Bacteroides were predominantly influenced by the oral butyrate treatment in the lean group, while Coriobacteriaceae and Clostridiales cluster XIVa were more affected in the metabolic syndrome group (77). Peripheral and hepatic insulin sensitivity improved only in lean individuals and not in individuals with metabolic syndrome, suggesting a potential difference in SCFA regulation of glucose metabolism between healthy individuals and individuals with metabolic syndrome. However, this study was a small pilot trial without a placebo group, highlighting the need for larger, follow-up, placebo-controlled studies investigating oral butyrate supplementations in humans.

De Groot et al. (78) also explored the effects of oral sodium butyrate capsules for 4 weeks in individuals with type 1 diabetes. They found decreased total fecal SCFA content with reduction in fecal acetate, propionate and butyrate levels along with no changes in HbA1c, fasting glucose, or daily insulin dose. These findings are in contrast with research performed in mouse models of type 1 diabetes (79–81). The reason for this discrepancy may lie in the mode of administration, dosage, length of the intervention, and the rate of disease progression.

Interestingly, several species of the Lachnospiracae family, which contains several butyrate-producing genera, were higher in abundance after butyrate supplementation (*L. pectinoschiza*, *D. formicigenerans*), while *Lachnospiracae*, *Blautia*, *Lachnospiracae Marvinbryantia* and *Lachnospiracae* NK4A136 group were less abundant and more prevalent after the placebo (78). It seems that oral butyrate generally induces a shift toward butyrate-producing bacteria in the microbiome.

Kjølbæk et al. (82) found that 4 weeks of arabinoxylan oligosaccharides supplementation had no effect on metabolic markers in individuals with metabolic syndrome. Nevertheless, the intervention did impact the gut microbiota composition, resulting in a bifidogenic effect (pronounced impact on the genus *Bifidobacterium*) together with an increased enrichment of butyrate producing bacteria such as *Eubacterium hallii*, *Faecalibacterium prautsnitzii*, and *Dorea longicatena*. The increase in bifidobacteria may lead to higher acetate and lactate production, which can then be metabolized by butyrate-producing species. This phenomenon is known as cross-feeding in which acetate and lactate can be used as an intermediate product that is further metabolized into butyrate by several bacterial species (83). Unfortunately, the study did not measure SCFA levels. Puhlmann et al. (84) observed similar findings after 3 weeks supplementation of dried chicory root supplementation in individuals with prediabetes. The most pronounced changes in microbiota composition were seen in the relative levels of *Bifidobacterium* and *Anaerostipes* spp. with an increase in fasting circulating acetate and a trend toward higher fecal butyrate levels compared to the control group. Although circulating butyrate remained unchanged in the chicory root supplementation group, it decreased more rapidly in the placebo group. They did report improvements in HOMA-IR, fasting insulin, and a slight decrease in fasting glucose within the intervention group but not compared to the placebo. Moreover, upon stratification into responders and non-responders, a significantly lower relative abundance of *Blautia* spp. was observed in responders, which translated into decreased HOMA-IR, fasting insulin and glucose levels compared to non-responders.

The remaining studies solely measured fecal butyrate levels. Mcintosh et al. (85) looked at the effect of a 4 weeks supplementation of whole-grain cereal foods compared to low-fiber refined-cereal foods on metabolic markers in men with overweight. Both the high-fiber rye and high-fiber wheat foods increased fecal butyrate levels, with rye showing a statistically significant difference compared to the placebo. Both foods improved 1 h postprandial glucose and insulin response after a test meal, which is in line with findings from acute human studies (11, 63–65) and an animal study involving rye bread (72). Hughes et al. (86) supplemented RS type 2 for 1 week in healthy individuals and similarly observed reduced 4 h postprandial glucose and insulin responses after a mixed breakfast meal. They did not find a change in fecal butyrate levels but observed increases in *Ruminococcus* and *Gemmiger* compared to the control group. When looking at correlations, *Faecalibacterium*, *Roseburia*, and *Ruminococcus* were positively associated with fecal butyrate (86), consistent with previous studies showing that fermentation of type 2 RS increases butyrate-producing species (87, 88). It is important to note that the study had a small sample size and a very short duration, and the correlations found were weak (86). Liu et al. (89) performed a cross-over study involving 2 weeks supplementation of fructo-oligosaccharide (FOS) and galacto-oligosaccharide (GOS) in healthy individuals. They reported decreased fecal butyrate levels and reductions in butyrate-producing species, along with higher glucose levels during an oral glucose tolerance test (OGTT) after FOS and higher fasting glucose after GOS supplementation. This may be due to interpersonal variability in OGTT responses and differences in individual microbiomes, as single prebiotics typically promote specific bacterial species (e.g., *Bifidobacterium* genus). Interestingly, the butyrate-producing species *Faecalibacterium*, *Ruminococcus*, and *Phascolarctobacterium* were strongly correlated with OGTT outcomes in the prediction model (89). Overall, factors such as high interpersonal variability, small sample size, short-duration and compliance should be considered when assessing the outcomes of the aforementioned studies (85, 86, 89). Moreover, differences in the types and doses of prebiotics used in studies might explain the varied response in glucose homeostasis. The relationship between different prebiotic sources and dosage should be further investigated.

Three studies have investigated the use of probiotics to modulate fecal butyrate levels and assess metabolic markers. Lee et al. (90) investigated the potential of different forms of oral *Bifidobacterium animalis* subsp. *lactis* supplementation in healthy individuals. They showed no alterations in fecal SCFA, plasma insulin, glucose and HOMA-IR following supplementation. Giljamse et al. (91) and Koopen et al. (92) investigated the effect of supplementing the strain *Anaerobutyricum soehngenii* in individuals with metabolic syndrome for 4 weeks. *Anaerobutyricum soehngenii* belongs to the Lachnospiracea family of the phylum Firmicutes and can convert sugars, lactate and acetate into butyrate. Both studies reported no difference in fecal butyrate and, overall, no effect on glucose homeostasis and insulin sensitivity. Nonetheless, Koopen et al. (92) administrated duodenal infusions of *Anaerobutyricum soehngenii* cells at week 0 and week 4 and observed a trend toward higher fecal butyrate levels. They also observed lower glucose variability within 24-h continuous glucose monitoring after the *Anaerobutyricum soehngenii* infusion with higher postprandial GLP-1 levels. Even though Giljamse et al. (91) reported no improvements in peripheral and hepatic insulin sensitivity, they did observe a positive correlation between fecal abundance of *Anaerobutyricum soehngenii* as well as with total fecal *Anaerobutyricum* spp. with insulin sensitivity. However, the three studies had a relatively small sample sizes, lacked control groups, and were of short durations (4 weeks), warranting further research to explore the long-term effects of probiotic supplementation and to better understand the mode of delivery (capsules, incorporated in dairy products), the doses, and underlying mechanisms of the different strains. Finally, one study studied the effect of a 2 weeks supplementation of either 50 or 100 mg/d riboflavin, a postbiotic, in healthy individuals for 2 weeks (93). Riboflavin acts as an electron mediator to promote the growth of *Faecalibacterium prausnitzii* (*F. prausnitzii*), a butyrate-producing bacterium (94), at low oxygen levels. They reported increased fecal butyrate levels with a trend toward lower postprandial insulin response and higher plasma GLP-1 levels between 15- and 120-min post-meal without changes in plasma glucose concentrations. Interestingly, the high dose of riboflavin (100 mg/d) did not affect the relative abundance of *F. prausnitzii* which could be due to the short-term supplementation and that *F. prausnitzii* is sufficiently present in the gut microbiota of healthy individuals. Nonetheless, riboflavin supplementation enhanced the bacterial network of *Faecalibacterium*, showing a positive correlation with fecal butyrate concentration and total SCFA levels (93). It would be interesting to investigate whether riboflavin modulates the gut microbiota and hence metabolic health in individuals with an impaired metabolic health.

The connection between butyrate levels (both plasma and fecal) and metabolic health appears to be less clear in short-term studies (Table 2). The limited number of studies measuring plasma butyrate complicates the understanding of its role in glucose homeostasis and insulin sensitivity, as fecal SCFA levels mainly reflect the balance between SCFA production, uptake and potential utilization by colonocytes (12). On the other hand, plasma butyrate can serve as a biomarker for microbial fermentation by the gut microbiota and may also act as a mediator of peripheral metabolic effects (95). Furthermore, the small size of the studies, variations in study population, and different types of interventions highlights the need for more targeted research to determine the most effective strategies for modulating the gut microbiome to enhance butyrate production, promote its release into circulation, and assess its specific effects on metabolic health.

### Long-term interventions

Fourteen studies examined the potential effect of an increased butyrate availability (as measured in plasma/serum and/or fecal levels) or enhanced microbial butyrate production on markers of glucose homeostasis over a more extended period, ranging from 6 weeks to 1 year (Table 2). Among these, nine studies have measured circulating butyrate levels. Wolever et al. (96) found that acarbose supplementation for 4 months led to increased serum butyrate levels and decreased mean 12 h glucose and insulin concentration following meal intake in individuals with impaired glucose tolerance. Acarbose functions as an α-glucosidase inhibitor, increasing the amount of dietary carbohydrates, primarily reducing the digestion of dietary starch, making it more available to the entire the colon for fermentation. Bell et al. (97) demonstrated that RS type 2 also raised both plasma and fecal butyrate levels after 6 weeks in individuals with type 1 diabetes. Additionally, circulating butyrate was inversely associated with HbA1c and the amount of insulin required to maintain stable blood glucose levels in the fasted state (basal insulin dose). Fecal butyrate levels were positively associated with the abundances of *Parabacteroides, B. longum* and *B. adolescentis*. Upadhyaya et al. (98) showed that a 12 weeks supplementation with RS type 4 flour increased fecal butyrate levels while reducing fasting glucose and HbA1c without impacting postprandial glucose in individuals with metabolic syndrome. Fecal butyrate levels were also correlated with the presence of *Ruminococcus lactaris* and *Oscillospira* spp., both known as butyrate-producing species. While *F. prausnitzii* did not increase significantly, it was negatively associated with the change in body fat percentage and body mass index (BMI). Additionally, the Firmicutes-to-Bacteroidetes ratio (F:B) decreased upon the RS type 4 treatment, which is found to be higher in individuals with overweight/obesity compared to lean individuals in some studies (99).

Freeland et al. (100) supplemented high-fiber wheat cereal compared to low-fiber cereal in individuals with hyperinsulinemia (≥40 pmol/L) for 1 year, monitoring 8 h metabolic profiles every 3 months. Mean plasma butyrate and plasma GLP-1 levels, adjusted for baseline, increased compared to placebo after 9 or 12 months. Additionally, there was a tendency for lower plasma glucose levels compared to placebo after 12 months. Two other studies that used whole-grain cereal (101) and inulin-propionate ester (102), found no changes in fasting plasma butyrate concentrations but observed a reduction in postprandial insulin response (101) and an improvement in insulin sensitivity (102). Ding et al. (103) replaced refined white rice with an equal portion of germinated brown rice, a rice type containing more whole grains and other nutritional components compared to white rice, for 12 weeks in individuals with T2DM. Germinated brown rice increased fecal butyrate levels, reduced fasting glucose, and showed a trend for a reduced HbA1c without affecting insulin levels. The prebiotic also increased levels of *Bifidobacterium* and *Butyricimonas* while decreasing *Prevotella*. These changes promoted intestinal barrier function and reduced inflammation, as observed by an improved balance of Th17 and Treg cells. Canfora et al. (104) supplemented GOS in individuals with prediabetes and overweight/obesity for 12 weeks. They found increased abundance of fecal *Bifidobacterium* with no changes in plasma and fecal SCFA levels, insulin sensitivity, or circulating metabolites such as glucose, GLP-1 and PYY. The lack of metabolic effects may be explained by the fact that GOS is quickly fermented in the cecum and the proximal part of the colon, whereas fermentation in the distal colon is associated with metabolic health benefits (11, 68, 69), as discussed earlier.

Zhao et al. (105) investigated the efficacy of a high-fiber diet including whole grains and prebiotics combined with traditional Chinese medicinal foods, compared to a control group receiving standard Chinese dietary recommendations for 12 weeks in individuals with TD2M. Both groups were administrated acarbose, an amylase inhibitor, as the standardized medication. They found that the intervention significantly increased fecal butyrate levels and the abundance of microbial pathways producing butyrate (increased gene richness). Furthermore, there was a decrease in HbA1c, fasting glucose and postprandial glucose, along with a higher fasting PYY levels and a greater postprandial AUC of GLP-1 at week 4. However, at week 12, only the HbA1c remained significantly different between the groups. Interestingly, they found that the response to the high-fiber intervention was related to the abundance of microbial SCFA-producing pathways. The abundance and diversity of high-fiber-promoted SCFA producers were higher in the intervention group and negatively correlated with multiple clinical parameters. Butyrate can be produced via four distinct pathways (4-aminobutyrate, acetyl-CoA, glutarate, and lysine) that contribute to butyrate production. Among these, the butyryl-CoA:acetate CoA-transferase gene, a key enzyme in the acetyl-CoA pathway, showed increased abundance only in the high-fiber diet group. Furthermore, the presence of *Lachnospiracae bacterium* showed significant associations with various metabolic outcomes, exhibiting positive correlations with the hormones PYY and GLP-1, while negatively correlating with markers including TNF-α, triglycerides, fasting blood glucose level, waist circumference, body weight, HbA1c, and HOMA-IR. They also observed that a subgroup of individuals, did not respond to the high-fiber intervention, along with no changes in the abundance of SCFA producing pathways, and some even showed worsened outcomes (105). This highlights the potential of personalized nutrition as a beneficial approach to manipulate the gut microbiota to manage metabolic disorders.

Palacios et al. (106) administrated multi-strain probiotic capsules or placebo for 12-weeks in individuals with T2DM. They found increased relative abundance of *Akkermansia muciniphila* and Bacteroidescaccae in the prebiotic group compared to placebo, with no changes in metabolic, inflammatory and permeability markers. However, after post-hoc analysis for metformin use, the subgroup taking metformin had a significant increase in plasma butyrate after probiotic supplementation compared to placebo (106). This was accompanied by a decrease in fasting glucose, HOMA-IR, and fasting plasma insulin. Moreover, plasma butyrate was negatively correlated with HOMA-IR, emphasizing the potential role of probiotics as an adjunct supplement to metformin to manage blood glucose levels. Additionally, participants on metformin showed increased relative abundance of, *Dorea formicigenerans*, *Dorea longicatena*, and Lacnospiraceae bacterium, with Lacnospiraceae bacterium showing a negative correlation to HbA1c. Perraudeau et al. (107) investigated two novel probiotics, containing either three or five distinct strains, for 12 weeks in individuals with T2DM. Both strains increased the relative abundance of *Akkermansia muciniphila* and *Bifidobacterium infantis* with no effect on fecal SCFA compared to the control. The three-strain probiotic resulted in no change in total glucose AUC_0-180min_ after a meal tolerance test (nutritional drink), HbA1c, or HOMA-IR. In contrast, the five-strain probiotic showed a decrease in total glucose AUC_0-180min_ but no changes in fasting glucose or HOMA-IR. More research is needed to better understand the role butyrogenic bacterial strains in glycemic control and to determine whether a more targeted synergistic approach could lead to improved health outcomes. Nevertheless, the latter study is the first randomized controlled trial in which multiple bacterial species were administrated as a probiotic to human subjects with T2DM (107). These findings can be supported by conducting studies with larger patient population, longer intervention periods, and the development of either new or different probiotic formulations.

Mueller et al. (108) investigated the effects of high-fiber diets with different macronutrient compositions (protein, carbohydrates, unsaturated fat) in individuals with overweight, obesity and (pre)-hypertension for 6 weeks using the cohort from the OmniHeart trial. Only the high protein diet, with primarily plant-based protein, significantly increased serum butyrate levels compared to baseline, unlike the carbohydrate and unsaturated fat rich diets. Conversely, when examining the mean change from baseline to the end of each 6-weeks diet period, elevated fasting serum butyrate levels were associated with higher fasting insulin and glucose levels. Vitale et al. (109) studied the acute and chronic effects (over 8 weeks) of a Mediterranean diet compared to the habitual diet in individuals with overweight/obesity. They found no changes in fasting butyrate levels but a significant increase in postprandial plasma butyrate after a Mediterranean lunch meal test in the Mediterranean group at 8 weeks. Furthermore, at the end of the intervention the postprandial butyrate response after a meal test at the end of the intervention was inversely correlated with postprandial plasma insulin response. Moreover, the dietary intake of fiber and the ratio of plant to animal protein were inversely associated with plasma glucose and insulin responses. The Mediterranean diet intervention also led to a rise in fiber-degrading bacteria, with elevated butyrate concentrations showing a positive correlation with the relative abundance of *Bacteroides xylanisolvens* and *Roseburia hominis*. In contrast, Meslier et al. (110) compared a Mediterranean diet to the habitual diet (isocaloric) over 8 weeks in individuals with overweight/obesity, finding no changes in fecal butyrate levels, blood glucose, plasma insulin, HOMA-IR, and GLP-1. They found an enrichment of members of *F. prausnnitzii*, *Roseburia* and members of the family taxa Lachnospiraceae in the Mediterranean group compared to control. Furthermore, participants in the quartile with the highest increase in fecal butyrate concentrations had higher levels of *F. prausnitzii* and *Lachnospiraceae* taxa after the intervention. When participants were stratified based on HOMA-IR change at 4 weeks compared to baseline, those who reduced their HOMA-IR on the Mediterranean diet exhibited a higher relative abundance of several *Bacteroides* species and lower levels of *Prevotella* spp. and *P. copri* compared to those whose HOMA-IR remained unchanged. This indicates interpersonal variability in gut microbiome, with some individuals harboring gut microbes more responsive to changes induced by the Mediterranean diet. These findings highlight the potential of using the initial gut microbiome to identify specific microbial phenotypes developing targeted nutritional interventions. In line, it has already been shown that specific metabolic phenotypes respond differently to dietary interventions (111–113). It is estimated that around 30% of participants do not respond or adhere to general population-based dietary guidelines (112, 114). The responsiveness appears to be associated with distinct metabolic or microbial phenotypes, potentially influenced by the host genome and its interaction with environmental factors, diet, and the gut microbiome. Moreover, insulin resistance can develop in different tissues, such as skeletal muscle, liver, and adipose tissue, each representing a different etiology toward T2DM and cardiometabolic diseases (112). Further tailoring dietary macronutrient composition according to tissue specific (e.g., liver, muscle) insulin resistance, as well as age, and sex-related differences, and microbial composition, can optimize precision nutrition.

Finally, seven studies that employed interventions to increase butyrate levels also measured inflammatory markers (98, 101, 103, 104, 106, 107, 110), of which only two studies reported a significant decrease in fasting proinflammatory cytokines such as IL-6, IL-8, LPS, TNF-α (Table 2). These two studies investigate the effects of supplementation with RS type 4 compared to control flour in individuals with metabolic syndrome (98), and the intake of whole grain germinated brown rice compared to refined white rice in individuals with T2DM (103), both using a 12-weeks intervention period. The supplementation with whole grain germinated brown rice also induced changes in the fecal microbiota composition, showing a negative association of *Roseburia intestinalis, Parabacteroides distasonis*, and *Eubacterium ramulus* with CD4+ T cells levels (103). The production of these bacterial strains is greatly influenced during gut dysbiosis in patients with inflammatory bowel disease, and they have been linked to both innate and adaptive immunological modulation (e.g., through the action of butyrate) (115). Additionally, Palacios et al. (106) noted a significant reduction in plasma zonulin levels after 12-weeks intake of the multi-stain probiotic capsules compared to placebo in individuals with T2DM. Zonulin is a marker of intestinal permeability, and butyrate has been shown to maintain gut barrier integrity, potentially reducing zonulin levels (116). In both studies (98, 103), increased fecal butyrate concentration were observed along with changes in the fecal microbiota composition and improved glucose homeostasis (reduced fasting glucose and insulin concentration). This suggests that butyrate may have a role in preserving intestinal homeostasis, which improve the regulation of inflammation and metabolism. However, more studies are needed to confirm this interaction in individuals with obesity and metabolic disturbances.

In summary, longer-term intervention studies were more successful in targeting microbial butyrate production, as reflected by enriched butyrate producers and increased plasma and/or fecal butyrate levels, compared to the short-term interventions (Table 2). However, the response in glucose homeostasis and insulin sensitivity varied among studies. While many studies reported increased abundances of butyrate-producing species and higher fecal and/or circulating butyrate levels, these changes did not consistently translate into improved metabolic outcomes (Table 2). Most studies primarily reported taxonomic shifts, with only a few providing evidence of changes in microbial functional pathways or metabolically relevant microbial activity. Contributing factors to the inconsistent findings regarding metabolic health may include small sample size, heterogeneity in study populations, and variability in the type and duration of interventions. While the use of prebiotics remains the most extensively studied and feasible option, additional research is necessary to identify the most effective approach to modulate the gut microbiome, the production and release of butyrate into the circulation, and improving metabolic health on a more personalized level.

## Discussion

**Figure 1:**
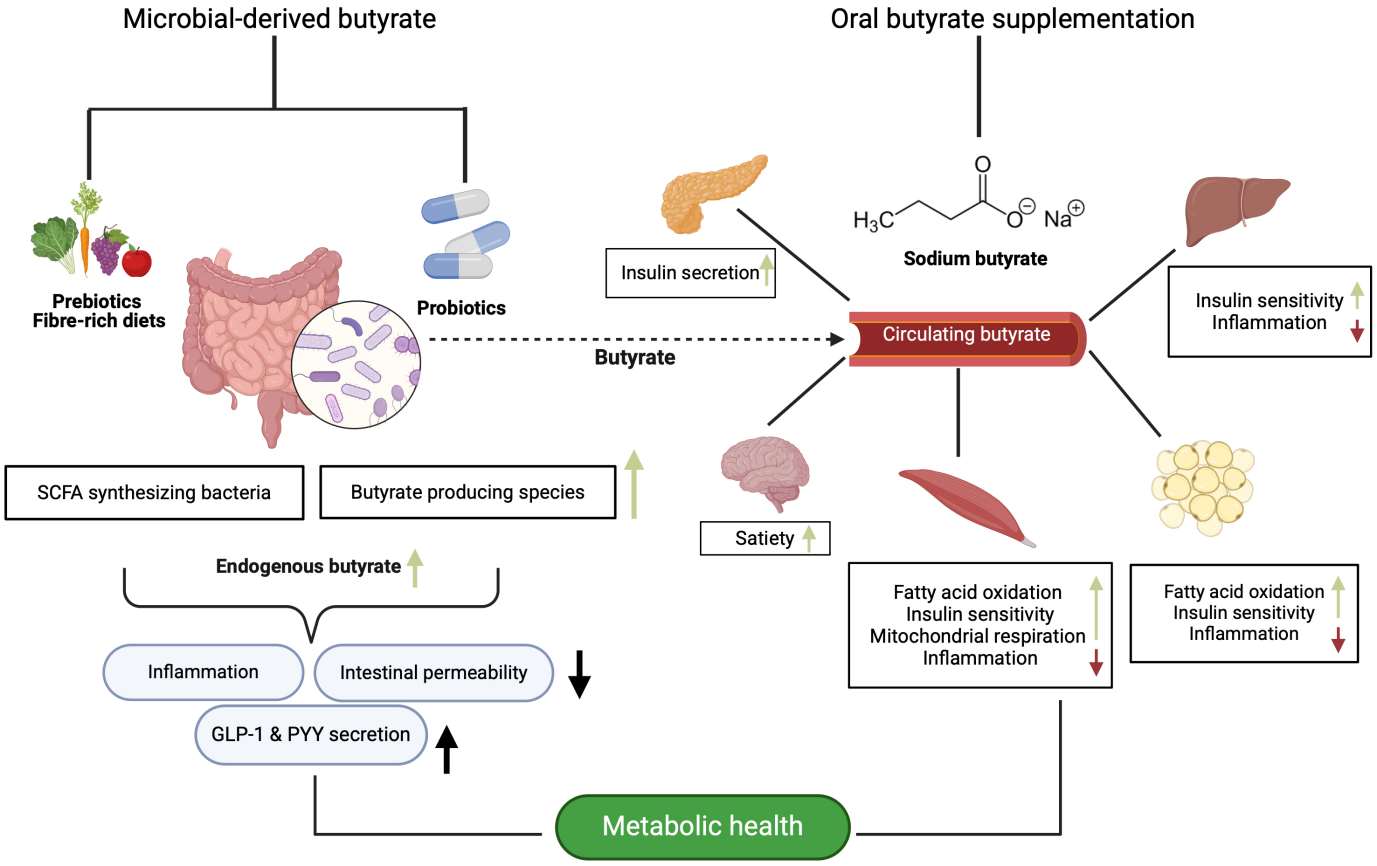
The potential metabolic effects of microbial-derived and butyrate supplementation on different organs, as evidenced primarily by animal and in vitro data. Created with BioRender.com.

As aforementioned, there is substantial evidence supporting the notion that butyrate plays a crucial role in gut and metabolic health though modulation of the gut microbiota. Serving as the primary energy source for colonocytes, butyrate not only maintains the integrity of colonocytes but also enhances the barrier function and may therefore regulate inflammatory process also in the periphery (15, 117, 118). Additionally, there is evidence suggesting that butyrate exerts effects beyond the intestine, influencing various metabolic processes in key metabolic organs such as the liver, adipose tissue and skeletal muscle (15, 117, 119). This paragraph contextualizes the findings of the clinical trials within the current literature focusing on implications of butyrate for metabolic (glucose homeostasis and insulin sensitivity) and intestinal health (Figure 1).

### Modulation of butyrate in intestinal and metabolic health

In reviewing 16 clinical studies that investigated the effects of pre-, pro-, or post-biotics on the gut microbiome (Table 2), nearly all reported either an increase in butyrate-producing species or a positive correlation between fecal butyrate levels and butyrate producers (77, 82, 84, 86, 91, 93, 97, 98, 103, 105–107, 110). Large cohort studies have associated butyrate-producing genera such as *F. prausnitizii*, *Eubacterium hallii*, *Oscillibacter*, and *Roseburia*, with enhanced insulin sensitivity and reduced dysglycemia in individuals with T2DM (16, 120–122). Consequently, a case–control study found reduced relative abundance of *Clostridiales, Akkermansia muciniphila*, and *Clostridum* in individuals with prediabetes, which exhibited a negative correlation between these taxa and fasting plasma levels of glucose, insulin, C-peptide, HOMA-IR, BMI, and waist circumference (122). Additionally, genetic analyses have revealed that increased gut butyrate production positively correlates with improved insulin responses during an OGTT (16, 123).

Nonetheless, the association between fecal butyrate levels and metabolic health remains ambiguous. Among the clinical studies (*n* = 16) reporting microbial changes in butyrate production, half observed effects on markers of glucose homeostasis and insulin sensitivity (Table 2). The intervention studies using RS (86, 97, 98, 103) demonstrated the most consistent effects on gut health and metabolic improvements associated with butyrate production. In general, RS fermentation has been demonstrated *in vitro* to produce the highest concentrations of SCFA and a greater ratio of butyrate to acetate (124, 125) with studies indicating that RS produces the highest amount of butyrate compared to other dietary fibers (126–128). Nevertheless, the relationship between the gut microbiome, diet and the host’s metabolism is intricate and multidirectional (13) and as indicated above, may vary between healthy and metabolically comprised individuals. Obesity is associated with reduced microbial diversity, a decrease in SCFA-producing bacteria, and altered SCFA concentrations (129).

Several studies, primarily involving Western populations, have reported elevated SCFA levels in the stool of individuals with obesity (130–132). One possible explanation for this finding is that the gut microbiota in obese individuals may lead to less efficient SCFA absorption, resulting in increased SCFA excretion (129). Consequently, individuals with overweight and/or metabolic impairments might exhibit reduced fermentation capacity compared to lean subjects (104, 133). Interindividual variability in the gut microbiota influences how dietary components, particularly dietary fibers, are metabolized into SCFA like butyrate (13, 134). This variability might affect the levels of butyrate produced, which plays a key role in metabolic health. As a result, differences in microbial composition can lead to varied impacts on host metabolism and the potential benefits of butyrate for improving glucose homeostasis and reducing metabolic disturbances. Diet-induced responses may depend not only on alterations in the gut microbiota composition, but also relates to metabolic phenotypes highlighting the need for optimal macronutrient composition tailored to each metabolic or microbial phenotype rather than a one-size-fits all approach (111, 112). For instance, three clinical studies (Table 2) have shown that individuals with obesity and prediabetes exhibit different responses to fiber interventions and oral butyrate compared to lean individuals (11, 77, 104). Altogether, these changes in microbial composition, activity, and metabolic dysfunction requires more targeted microbiome modulation through personalized nutrition. This involves detailed characterization of both the baseline gut microbiota composition/functionality and metabolic phenotype to effectively manage T2DM and potentially other dysbiosis-related diseases.

Another important aspect when examining the role butyrate in metabolic health is that it is hard to measure butyrate production or dynamics and that the site of colonic fermentation and thus butyrate production may differentially affect metabolic outcomes. Previous research showed that circulating, not fecal, SCFA are related to insulin sensitivity, lipolysis and GLP-1 concentrations in humans (135). Fecal SCFA concentrations are not a reliable indicator of their actual levels or production rates within the intestine, as the majority (around 95%) of colonic SCFA are absorbed the host, with only about 5% excreted in feces (52). These concentrations can be influenced by factors such as intestinal transit, permeability, metabolite transportation, and sample handling (136). Consequently, fecal SCFA levels serve more as a proxy for the net balance between colonic production and absorption. Due to their variability across different populations and limited reflection of *in vivo* colonic fermentation, fecal SCFA offers limited insights about actual intestinal SCFA metabolism, systemic bioavailability, and potential metabolic impacts. For example, fecal acetate and butyrate did not correlate with their respective circulating concentrations, indicating different levels in the circulation (135). In contrast, circulating fasting butyrate levels were positively associated with fasting GLP-1 concentrations and inversely associated with fasting glucose concentration and BMI (135). Zhang et al. (137) found that plasma butyrate concentrations were negatively associated with BMI, body fat percentage, visceral fat area, and 2 h plasma glucose after an OGTT in individuals with T2DM and obesity (137). Interestingly, when looking at the clinical trials (Table 2), eight studies (11, 64, 65, 74, 76, 96, 106, 109) reported both an increase in plasma butyrate and beneficial changes in glucose homeostasis, mostly though dietary fiber intervention. The site of SCFA production in the colon matters for metabolic health benefits (11, 69, 70, 138). SCFA are released in higher quantities from the distal colon, up to ~3 times more for butyrate, compared to the proximal colon (70). This could be due to the mucosa of the proximal intestines already metabolizing a significant portion of the produced SCFA (70). Additionally, SCFA released from the distal colon partly bypass the liver, thereby directly reaching the systemic circulation (11, 69, 70). Other distinctions between the proximal and distal colon include variations in the expression of GPRs and differences in microbiota composition and activity, leading to increased microbial utilization in the distal colon (11, 69, 70). Therefore, increased fermentation in the distal colon (e.g., from slowly fermentable fibers) may have a higher greater potential to positively impact host metabolism. The liver is the major organ taking up all the SCFA from the circulation in which butyrate regulates hepatic insulin sensitivity, fat deposition and inflammation (15, 70, 139). However, only a minor fraction of the colon-derived butyrate is transferred to the systemic circulation to be taken up by the peripheral tissues (15, 117, 140). Therefore, further optimization of longer-term dietary interventions aimed at promoting butyrate production in the distal colon is warranted to effectively enhance metabolic health. For example, a high fiber diet rich in RS, or indirectly increasing the amount of starch delivered to the colon (e.g., acarbose), may represent an appealing approach to enhance the gut microbial environment, indirectly improving host metabolic functions. Ultimately, further research involving larger sample sizes and more diverse disease cohorts is needed to clarify the role of circulating butyrate in obesity and metabolic disorders. Such insights could support the development of precision nutrition strategies tailored to individual baseline microbial composition, functional capacity, and metabolic phenotype.

### Mode of butyrate administration

The mode of administration is also important to consider when studying the impact of butyrate on metabolic health. Majka et al. (31) compared 4-weeks of direct butyrate (NaBut) supplementation with butyrate produced from fiber (β-glucan) fermentation in young male mice (C57BL/6J) fed a HFD. Oral butyrate supplementation was more effective in preventing body weight gain by reducing epididymal white adipose tissue mass and saturated lipid accumulation, in adipose tissue, while fiber supplementations more effectively modulated the gut microbiome, increasing bacterial diversity and SCFA producers. Unfortunately, they did not assess any markers related to the glucose homeostasis. The question remains as to which type of supplementation will provide more balanced and long-term health benefits by targeting diverse tissues.

Oral butyrate supplementation, commonly in the form of NaBut (salt of butyric acid), may be a promising strategy for directly influencing the peripheral tissues. Another form is butyric acid esters, known as butyrins, which are short-chain glycerides consisting of butyric acid molecules attached to a glycerol backbone. The most common structure among butyrins is tributyrin, a trimolecular lipid consisting of butyrate esterified with glycerol (141). Both butyric salts and butyrins present certain limitations that impede their clinical practice. NaBut has low bioavailability as it rapidly absorbed in the upper gastrointestinal tract (21, 141, 142), with free butyrate quickly being cleared from the circulation (21, 142). Although various encapsulation techniques have been employed to delay butyrate release in the upper gastrointestinal tract, NaBut has still an offensive odor and flavor making it unpleasant to handle and consume. Furthermore, the elevated sodium content might trigger increased thirst and blood pressure, as both are responsive to rises in plasma sodium concentrations (143, 144).

Butyrins require lipase activity to release butyrate resulting in more favorable pharmacokinetics, but it remains to be at least in part rapidly absorbed in the stomach. While tributyrin is better tolerated than NaBut, it can still cause side-effects such as fatigue, nausea and vomiting (142). To delay the release of butyrate, incorporating butyrins into emulsions containing long-chain fatty acids could beneficially alter the structure, resulting in a delayed release of butyrate from the glycerol molecules. This concept has been tested by van Deuren et al. (145), in which they compared *in vitro* the release of SCFA from tributyrin to an oil containing butyrate and hexanoate esterified with long chain fatty acids. While tributyrin was rapidly cleaved in the stomach resulting in a high release of SCFA, the majority of SCFA remained esterified to glycerol in the small intestine when using the triglyceride oil.

Moreover, the amount of butyrate reaching the intestine from current butyrate supplements may be inadequate to elevate serum butyrate concentrations for therapeutical effects. In cases of intestinal damage or inflammation, colonocytes undergo increased metabolism, which, along with changes in microbial composition, can result in reduced endogenous butyrate production (146). To compensate, multiple capsules would need to be consumed to achieve a physiological level of butyrate in the circulation. Further research is needed to determine the effective butyrate doses in individuals with metabolic and gut microbiome disturbances. Additionally, more human studies are needed to investigate whether oral butyrate supplementation increases circulating butyrate levels and how sustained elevated circulating butyrate concentrations translate into potential long-term metabolic effects. Other strategies to optimize butyrate supplementation include modulating the release of butyrate via special matrices and microencapsulation techniques and masking the unpleasant smell and taste of butyrate to enhance the edibility and palatability.

Newer methods to supplement butyrate include postbiotics and acetylated starches. Postbiotics are defined as preparations of inanimate microorganisms and/or their components that confer a health benefit on the host, representing a new category of biotics such as vitamins, enzymes and exopolysaccharides (147). For butyrate, most probiotics are currently not considered safe for human consumption. However, butyrate-rich postbiotic preparations that are safe for human use can be made using butyrate-producing bacteria (147). Another approach could be incorporating lactic acid bacteria into vegetable-, milk-, and cereal-based fermented foods. Lactic acid bacteria are gram-positive bacteria that yield upon fermentation high levels of lactate and acetate, which can have a positive downstream effect on butyrate and propionate concentrations in the colon through cross-feeding (147).

Furthermore, animal studies have shown that acylated starch can deliver specific SCFA to the large intestine, with butylated starch successfully reaching the colon (148–150). Acylating starch with different SCFA can beneficially modify the starch, resulting in reduced digestibility, increased RS content, and additional SCFA delivery beyond what is produced through fermentation (151, 152). Although it’s impact on glucose and lipid metabolism regulation remains unclear, Li et al. (151) found that acylated starch had a greater impact on body weight loss, fasting blood glucose, and serum insulin levels compared to native RS in a TD2M rat model. Additionally, the acylated starch enhanced butyrate-producing bacteria, such as *Coprococcus* and *Butyricimonas*, consistent with higher concentrations of both fecal and plasma butyrate levels. Nonetheless, both methods are still emerging, and further research is needed to first better understand the uptake of SCFA in the small intestine and the extent to which butyrate-rich preparations can reach the large intestine in humans. Table 3 provides an overview of the different supplementation methods along with their respective advantages and disadvantages.

**Table 3:** Comparison of different modes of butyrate supplementation methods: pros and cons.

Supplementation method	Pros	Cons
Sodium butyrate	High dissolution rate High biological activity	Very low bioavailability → low physiological level of butyrate Unpleasant taste and odor Elevated sodium content
Butyrins	More favorable pharmacokinetics compared to NaBut	Rapid absorption in the stomach Low bioavailability → low physiological level of butyrate Potential side effects, e.g., fatigue, nausea, vomiting
Prebiotics (e.g., RS)	Encourages natural butyrate production Promotes overall gut health	Effectiveness depends on individual microbiota Potential initial digestive discomfort Reduced fermentation capacity in individuals with overweight and/or metabolic disturbances
Acylated starch	Targeted delivery to large intestine Enhances RS content	Complex production process Long-term effects not well understood Potential digestive side effects
Postbiotics	Potentially safer than live probiotics	Research is in its early stages Need for better understanding of SCFA uptake and effects

## Future perspectives

Oral butyrate supplementation in animal studies has demonstrated promising effects on intestinal and metabolic health. Given that individuals with disturbances in glucose homeostasis often have a lower butyrate-producing potential of the gut microbiota (17–20), replenishing either the levels of butyrate or butyrate producers could serve as a therapeutical strategy to prevent T2DM. However, using butyrate as a therapeutic agent presents several challenges. Most human clinical trials have primarily focused on oral butyrate supplementation in intestinal disorders. Of the 35 trials identified with the search string, 26 investigated the effect of butyrate on glucose homeostasis in individuals with overweight/obesity and a form of metabolic disorder. The outcomes were mixed, with no consensus on whether higher butyrate levels (plasma and/or fecal) or enriched butyrate-producing species, achieved through oral butyrate supplementation or via pre- and post-biotics, led to improvements in glucose homeostasis and/or insulin sensitivity. Furthermore, it is challenging to determine whether increased microbial butyrate production truly improves the metabolism of peripheral tissues such as liver, skeletal muscle and adipose tissue since as the extent to which butyrate reaches the circulation is uncertain. Butyrate is primarily absorbed and metabolized by colonocytes followed by hepatocytes (rapid hepatic clearance), with only minimal amounts entering the circulation to reach peripheral tissues (15, 153). However, butyrate in the gut may stimulate the release of incretins PYY and GLP-1, which, through signaling pathways, could enhance peripheral metabolism (11, 15, 68, 69). Although there is strong evidence linking butyrate production to metabolic improvements, causality remains uncertain due to inconsistent findings in intervention studies. Since the distal colon releases a higher quantity of SCFA than the proximal colon (70), increasing availability or microbial production of butyrate in this region may help maintain systemic levels while also supporting gut signaling. Techniques for measuring butyrate in feces and blood may not accurately represent its dynamics, as SCFA are very volatile, and considerable variation exists in the levels of butyrate among individuals with metabolic conditions such as T2DM (14). To gain a better understanding of butyrate kinetics, future research could employ isotopic tracers to trace the origin and absorption of butyrate. Additionally, utilizing indigestible electronic capsules to monitor microbial fermentations directly in the gut and using techniques such as flow cytometry for more live-detection of butyrate-producing bacteria and or pathways could offer valuable insights. Another aspect is that the mechanistic evidence of butyrate is mainly driven from animal models and *in vitro* work. While mouse models are commonly used to investigate host–microbe interactions, several limitations should be acknowledged when translating these findings to humans. Although human and mice share some anatomical similarities and common bacterial genera in the gut, they differ significantly in body size, metabolic rate, diet, and microbial composition abundances as well as functionally (154). In addition, much of the microbiota research is conducted using the same inbred wild-type mouse strains, with limited testing across other strains that may harbor distinct microbial communities. External factors such as the provider and the conditions of the rearing facility can also influence the gut microbiota composition in mice (154). Furthermore, significant differences in gut-derived butyrate kinetics exist between rodents and humans, driven by species-specific variations in colonic and hepatic metabolism, as well as in the expression of transporters and metabolic enzymes (155). These factors affect butyrate bioavailability and constrain the direct translation of rodent findings to human physiology.

*In vitro* studies often apply potential supra-physiological concentrations of butyrate with range of different experiment settings making it hard to adequately address the impact of butyrate on glucose homeostasis, insulin sensitivity and inflammatory processes. Furthermore, for both types of research, it is challenging to distinguish whether the reported effects arise directly from the influence of butyrate on target tissues or indirectly via signaling pathways, such as the stimulation of the secretion of GLP-1 and PYY by enteroendocrine cells. Comparing metabolic markers of animals on a HFD receiving oral butyrate and intravenous butyrate at equivalent doses could help in elucidating the route of mechanisms of butyrate. The beneficial effects of butyrate likely stem from its ability to improve gut barrier function, reduce inflammation, and enhance peripheral tissues, all of which contribute to better metabolic health. This complexity makes it challenging to pinpoint butyrate’s exact role, suggesting that either direct supplementation or indirect targeting through the microbiome might be effective strategies to harness its benefits.

In addition to technical and mechanistic considerations, larger-scale placebo-controlled randomized trials with longer intervention periods are necessary to determine whether increasing circulating butyrate is an effective target for metabolic disorders. Extended intervention periods are essential to allow sufficient time to increase systemic butyrate concentrations to affect gene expression and cellular processes in peripheral metabolically active organs such as the adipose tissue or skeletal muscle. Moreover, the response to dietary interventions could be more by incorporating a combination of butyrate-producing fibers (e.g., resistant starch, arabinoxylan, wheat, rye), taking into account the initial microbiota composition, functionality, and metabolic phenotype. This could ensure tailored responses as there are still non-responders to high-fiber interventions resulting in different degrees of altered microbiome composition and SCFA metabolism (105, 106, 109, 110). Lastly, further optimization of butyrate supplements in terms of dosage, bioavailability, and palatability to ensure delivery to the large intestine, coupled with measuring circulating butyrate levels, will help in determining the optimal approach to sufficiently increase butyrate delivery and uptake by peripheral tissues.

## Concluding remarks

Butyrate plays a crucial role as a mediator in the regulation of host metabolism by modulation of microbial production. In animal and cell-based models, butyrate attenuates both the microbial dysbiosis and metabolic disturbances caused by a HFD, via various mechanisms including HDAC inhibition, mitochondrial and skeletal muscle adaptations, and reducing inflammation.

Despite these promising findings in preclinical models, several challenges must be addressed before butyrate can be effectively used as a therapeutic target (e.g., in the form of supplement) for preventing and/or treating metabolic disorders. In humans, the connection between increased plasma butyrate and improved glucose homeostasis seems to be more evident in metabolically healthy individuals than in those with obesity and/or metabolic disturbances. More specific research is warranted to gain a deeper understanding of the precise mechanisms though which butyrate impacts gut health and metabolic control. This includes investigating how these mechanisms differ between healthy individuals and those with metabolic disturbances. Additionally, it is essential to understand how microbial production of butyrate and its absorption by the host differ across distinct metabotypes or microbial phenotypes, and how we can develop optimal strategies for supplementing butyrate to prevent the development of obesity and obesity-related complications. Oral butyrate supplements may be beneficial in specific cases where dietary changes alone are insufficient to achieve important metabolic health improvements.
