# Supplementary material for: The impact of butyrate on glycemic control in animals and humans: a comprehensive semi-systemic review
Source: Front Nutr. 2025 Jun 10;12:1603490. doi: 10.3389/fnut.2025.1603490 (PMC12185432; doi:10.3389/fnut.2025.1603490)
Supplement: Supplementary file 1 [file Table_1.DOCX]

**Supplementary**

**Table 1**: PICOS criteria for inclusion and exclusion of studies.

| **Parameter** | **Inclusion criteria** | **Exclusion criteria** |
| --- | --- | --- |
| Participants | Healthy adults  Adults with:   - Overweight/obesity - Diabetes (type 1 and type 2) - Prediabetes (e.g., impaired glucose tolerance) - Metabolic syndrome - Hyperinsulinemia | Inflammatory bowel disease Colon cancer  Cardiovascular disease  Hypertriglyceridemia  Dementia |
| Interventions | Butyrate supplementation (oral/IV infusion/enema of butyrate)  Dietary fibre(s) supplementation and/or high fibre diets (e.g., Mediterranean)  Butyrogenic strains supplementation | Faecal transplantation |
| Comparators | Placebo vs intervention (endpoint); baseline vs endpoint (intervention group) | Intermediate time points  No baseline data |
| Outcomes | Concentrations of fasting insulin, fasting glucose, or HOMA-IR as well as reporting of changes in plasma or faecal levels of butyrate at the end of intervention  Markers of butyrate microbiome activity such as enriched pathways or species | Studies not reporting concentrations of fasting insulin, fasting glucose, or HOMA-IR (e.g., AUC, regression/correlation coefficients, bar graphs, difference in concentrations) and studies not reporting the change in either butyrate concentration or microbiome activity |
| Study design | Randomized clinical trials, crossover studies, or treatment-only studies | Non-original papers (reviews, conference reports, clinical trial protocols) and non-English papers |

Abbreviations: AUC, area under the curve; HOMA-IR, homeostatic model assessment of insulin resistance; IV, intravenous.
